# Facial age affects emotional expression decoding

**DOI:** 10.3389/fpsyg.2014.00030

**Published:** 2014-02-04

**Authors:** Mara Fölster, Ursula Hess, Katja Werheid

**Affiliations:** ^1^Clinical Gerontopsychology, Department of Psychology, Humboldt-Universität zu BerlinBerlin, Germany; ^2^Social and Organizational Psychology, Department of Psychology, Humboldt-Universität zu BerlinBerlin, Germany

**Keywords:** emotional facial expressions, facial expression decoding, older face, aging, own-age advantage, response bias, expressivity

## Abstract

Facial expressions convey important information on emotional states of our interaction partners. However, in interactions between younger and older adults, there is evidence for a reduced ability to accurately decode emotional facial expressions. Previous studies have often followed up this phenomenon by examining the effect of the observers' age. However, decoding emotional faces is also likely to be influenced by stimulus features, and age-related changes in the face such as wrinkles and folds may render facial expressions of older adults harder to decode. In this paper, we review theoretical frameworks and empirical findings on age effects on decoding emotional expressions, with an emphasis on age-of-face effects. We conclude that the age of the face plays an important role for facial expression decoding. Lower expressivity, age-related changes in the face, less elaborated emotion schemas for older faces, negative attitudes toward older adults, and different visual scan patterns and neural processing of older than younger faces may lower decoding accuracy for older faces. Furthermore, age-related stereotypes and age-related changes in the face may bias the attribution of specific emotions such as sadness to older faces.

## Introduction

Facial expressions convey important information on emotional states of our interaction partners (Ekman et al., 1982). Thus, the correct interpretation of facial expressions may facilitate emotional understanding and enhance the quality of interpersonal communication.

Recent evidence suggests that the correct interpretation of emotional expressions may be negatively affected in older age due to processes related to both the sender and the observer. The majority of the extant research has focused on the influence of the observers' age and concludes that older observers have deficits in the decoding of specific emotions (see Ruffman et al., 2008; Isaacowitz and Stanley, 2011, for reviews).

The age of those showing the facial expressions was initially less often considered. As a possible reason, the influential model of face processing by Bruce and Young (1986) postulated that the decoding of facial expressions is robust to the idiosyncratic features of a given face, which might be influenced by age, sex or other factors. However, this proposition has been subject to controversial debate (e.g., Schweinberger and Soukup, 1998; Schyns and Oliva, 1999; Kaufmann and Schweinberger, 2004; Calder and Young, 2005; Aviezer et al., 2011; Barret et al., 2011). Instead, it is likely that wrinkles, folds and the sag of facial musculature in the older face affect the interpretation of facial expressions. This assumption has been confirmed by recent results of decoding accuracy varying with the age of the face (Malatesta et al., 1987b; Borod et al., 2004; Ebner and Johnson, 2009, 2010; Murphy et al., 2010; Richter et al., 2011; Riediger et al., 2011; Hess et al., 2012; Ebner et al., 2012, 2013; Hühnel et al., 2014). Previous reviews have mainly focused on the age of the observer (Ruffman et al., 2008; Isaacowitz and Stanley, 2011) or on age-of-face effects on face identity recognition (e.g., Rhodes and Anastasi, 2012). Adding to this work, the present review is the first to focus on the influence of facial age on expression decoding and its possible underlying mechanisms, taking into account most recent work on this subject published after these previous reviews. Our aim was to compile and evaluate findings, thereby focusing on the question to which extent methodological differences between studies may account for inconsistent results. Further, we wish to identify unresolved research questions and suggest topics for future research. We will first give a very brief overview of the influence of the observers' age on decoding accuracy and the mechanisms underlying these effects. However, as this research has already been reviewed elsewhere (Ruffman et al., 2008; Isaacowitz and Stanley, 2011), we will mainly focus on the influence of the faces' age.

## Influence of the observers' age

**Figure 1:**
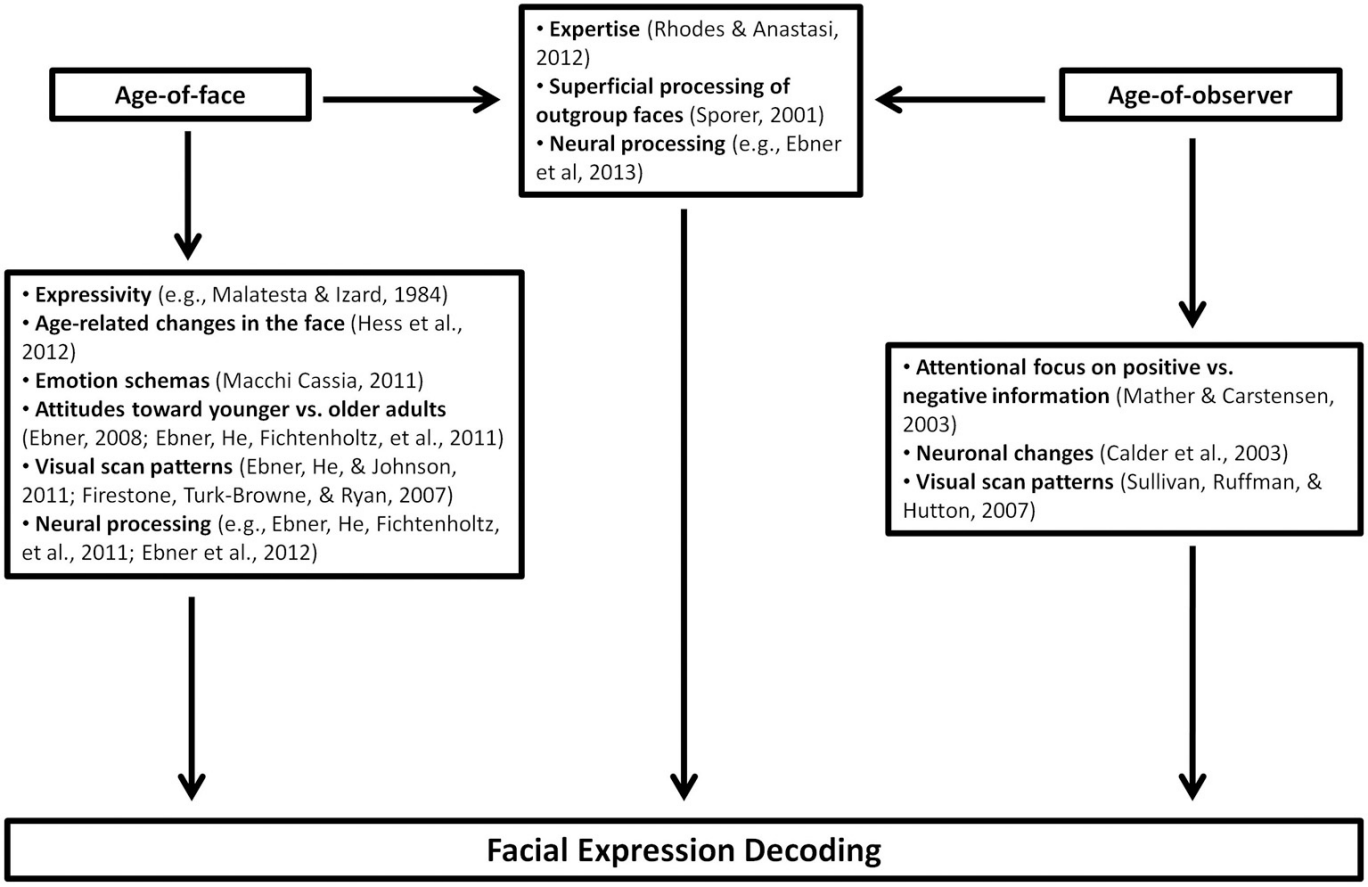
**Overview of mechanisms underlying effects of the age of the face (left part) and the age of the observer (right part) as well as own-age effects (central part) on facial expression decoding**.

An age-related decline in decoding facial expressions has been repeatedly reported (e.g., Calder et al., 2003; Ruffman et al., 2008; Isaacowitz and Stanley, 2011). However, recent evidence suggests that this decline is confined to specific emotions. An overview of mechanisms underlying emotion-specific effects of the observers' age on facial expression decoding is given in Figure 1 (right part). Some studies found an age-related deficit in decoding negative, but not positive emotional expressions (Phillips et al., 2002; Williams et al., 2006; Keightley et al., 2007; Ebner and Johnson, 2009). In addition, older observers had a greater bias toward thinking that individuals were feeling happy when they were displaying either enjoyment or non-enjoyment smiles (Slessor et al., 2010; but see Riediger et al., under review). These results have been accounted for by an information processing bias by older observers, leading to increased attention toward positive compared to negative information (Mather and Carstensen, 2003). This explanation is based on the socioemotional selectivity theory (SST, Carstensen and Charles, 1998), stating that older persons are, due to their limited future time perspective, inclined to engage in tasks related to emotional balance and well-being. Younger persons, in contrast, favor information seeking over emotionally rewarding goals and, thus, may be more inclined to attend to other persons' negative emotional states (Carstensen and Mikels, 2005). However, Isaacowitz and Stanley (2011) argued that the preserved ability to decode happiness may as well be due to the relative ease of the task, when happiness is the only positive response option. Supporting this assumption, age effects for positive emotions emerged when the task was more difficult (Isaacowitz et al., 2007). Further evidence against the SST-based account is that the majority of research on emotional prosody and body language suggests that older observers have difficulties to decode positive as well as negative emotions (emotional prosody: Taler et al., 2006; Ruffman et al., 2009a,b; Lambrecht et al., 2012, body language: Ruffman et al., 2009a,b, but see Montepare et al., 1999, for an exception).

Further conflicting the SST-based account, some studies found an age-related improvement in decoding disgust, together with no age differences for happiness and an age-related deficit in decoding sadness (Suzuki et al., 2007), or anger, fear and sadness (Calder et al., 2003). There are two alternative explanations for these findings.

The first explanation is based on observed age differences in visual scan patterns: older observers focus primarily on the lower part of the face and neglect the upper part (Wong et al., 2005; Sullivan et al., 2007). As the upper part plays a more important role for expressions of anger, fear and sadness, but not for disgust and happiness (Calder et al., 2000), this may explain why older observers are especially impaired in decoding these emotions. In line with this explanation, older observers' poor performance for decoding anger, fear and sadness correlated with fewer fixations to the top half of faces (Wong et al., 2005). However, Ebner et al. (2011c) found that visual scan patterns were independent of the observers' age, but rather varied with the expression. Thus, evidence for age differences in visual scan patterns is mixed. Furthermore, as already mentioned above, older observers' difficulties are not restricted to decoding facial expressions, but also emerge when decoding emotional prosody and bodily expression, at least rendering visual scan patterns as sole underlying mechanism unlikely.

The second explanation states that the brain regions that are responsible for decoding emotions, differ between the various emotions and that these regions are also differently affected by age-related changes (Ruffman et al., 2008; Ebner and Johnson, 2009; Ebner et al., 2012). As the frontal region, which is especially important for anger and sadness (Murphy et al., 2003), and the amygdala, which is important for fear (Murphy et al., 2003; Adolphs et al., 2005), are particularly affected by age-related changes (Jack et al., 1997; Bartzokis et al., 2003), a stronger age-related decline in decoding these emotions is predicted. In contrast, the basal ganglia, playing an important role for disgust (Phan et al., 2002), are less strongly affected by age-related changes (Raz, 2000; Williams et al., 2006), possibly resulting in a relatively preserved ability to decode disgust. Wong et al. (2005) further suggested that age-related declines in the frontal eye field, an area in the frontal lobe, may lead to deficits in visual attention, possibly leading to dysfunctional visual scan patterns. In addition, recent fMRI studies investigating brain activity in younger and older observers while viewing emotional faces suggest functional brain changes with age (Williams et al., 2006; Keightley et al., 2007). Although the pattern of results is somewhat mixed, older observers showed less amygdala activation (Gunning-Dixon et al., 2003; Fischer et al., 2005, 2010), but more prefrontal cortex activation (Gunning-Dixon et al., 2003; Fischer et al., 2010) compared to younger observers when viewing emotional faces. Fischer et al. (2005) suggested that this may represent an attempt to compensate for diminished functions in other brain regions than the frontal brain. Williams et al. (2006) further argued that this may reflect a shift from automatic processing to a more controlled processing of emotional information, possibly enabling older observers to better selectively control reactions to negative stimuli and finally leading to better emotional well-being. However, it may also be important to consider the valence of the facial expressions, and to differentiate between dorsomedial (dmPFC) and ventromedial prefrontal cortex (vmPFC). A recent study (Ebner et al., 2012) suggests that the vmPFC is more involved in affective and evaluative processing, whereas the dmPFC is involved in cognitively more complex processing. This functional dissociation seems to be largely comparable between younger and older observers. However, older observers showed increased dmPFC activity to negative faces, but decreased dmPFC to positive faces, possibly representing more controlled processing of negative compared to positive faces in older observers (Ebner et al., 2012).

## Influence of the faces' age

Concerning the age of the face, the majority of previous research found that posed emotional facial expressions were decoded less accurately in older compared to younger faces, irrespective of the target emotion (Borod et al., 2004; Riediger et al., 2011; Ebner et al., 2012, 2013) or with the exception of happiness (Ebner and Johnson, 2009; Ebner et al., 2011c), which may be due to ceiling effects. Hess et al. (2012) confirmed this finding with artificially created face stimuli displaying identical expressions for younger and older faces. As an exception, Ebner et al. (2010) found no age difference for posed fear expressions, but for happiness, anger, sadness, disgust, and neutrality expressions. Taken together, these results suggest that decoding emotional expressions is more difficult in older compared with younger faces. However, results obtained with spontaneous, dynamic expressions yielded a more heterogeneous pattern of results. Whereas Richter et al. (2011) confirmed the generally lower decoding accuracy for older faces, and Murphy et al. (2010) found a more accurate differentiation between posed and spontaneous dynamic smiles in younger than older faces, Riediger et al. (under review) found no main effect of facial age on the differentiation between spontaneous and posed dynamic smiles, Malatesta et al. (1987b) found no significant age difference on decoding emotional facial expressions and Hühnel et al. (2014) found even higher decoding accuracy for older faces displaying sadness. In the following, we will discuss possible mechanisms underlying these results. An overview of these studies is given in Table 1 and an overview of the underlying mechanisms is given in Figure 1 (left part).

**Table 1:** **Summary of research on age-of-face effects on decoding accuracy**.

**Authors**	**Age-of-face**	**Stimuli description/database**	**Target emotions**	**Observers**	**Dependent variables**	**Main age-of-face results**
Borod et al., 2004	30 YA, 30 MA, 30 OA, only females	Still posed expressions, developed for this study	Happiness, pleasant surprise, sadness, disgust, neutrality	12 YA, 12 MA, 12 OA, only females	Multiple choice emotion identification Confidence of rating	OA faces were rated less accurately and with less confidence than YA faces No own-age effect
Ebner et al., 2011c	8 YA and 8 OA per emotion	Still posed expressions [FACES developed by Ebner et al., 2010]	Happiness, anger, fear, sadness, disgust, neutrality	30 YA, 30 OA	Multiple choice emotion identification Visual scan patterns	Higher accuracy for YA than OA faces for anger, fear, disgust, sadness and neutrality No age-of-face effect for happiness No own-age effect Both YA and OA looked longer at own-age than other-age faces Longer looking at own-age faces predicted better own-age expression identification
Ebner and Johnson, 2009	8 YA and 8 OA per emotion	Still posed expressions [FACES developed by Ebner et al., 2010]	Happiness, anger, neutrality	32 YA, 24 OA	Multiple choice emotion identification Self-reported contact to YA and OA	Higher decoding accuracy for YA than OA faces for anger and neutrality No age-of-face effect for happiness No own-age effect The more contact observers reported with the own age group, the less they were able to identify expressions of the other age group No relationship between own-age contact and own-age decoding accuracy
Ebner et al., 2012,^1^	16 YA and 16 OA per emotion	Still posed expressions [FACES developed by Ebner et al., 2010]	Happiness, anger, neutrality	30 YA, 32 OA	Multiple choice emotion identification fMRI data	Higher accuracy for YA than OA faces No age-of-face by emotion interaction No own-age effect Functional dissociation between vmPFC (affective processing) and dmPFC (cognitive control) Greater dmPFC activity for OA compared to YA faces
Ebner et al., 2010	58 YA, 56 MA, 57 OA	Still posed expressions (development and validation of FACES in this study)	Happiness, anger, fear, sadness, disgust, neutrality	52 YA, 51 MA, 51 OA	Multiple choice emotion identification Age estimation	Higher accuracy for YA than OA faces for happiness, anger, sadness, disgust, and neutrality No age-of-face effect for fear No own-age effect Happy faces were perceived as younger and fearful faces as older than faces with other expressions
Hess et al., 2012	6 YA, 6 OA	Morphed identical facial expressions to neutral faces from Minear and Park (2004)	Happiness, anger, sadness	65 YA	Continuous emotion rating	OA faces were rated as more intense on inaccurate emotions, but as less intense on accurate emotions than YA faces
Hühnel et al., 2014	4 YA, 4 OA	Videos of adults talking about biographic episodes (without sound)	Happiness, anger, sadness, disgust	39 YA, 39 OA, only females	Continuous emotion rating Mimicry measured via facial EMG	Higher accuracy for YA than OA faces for happiness and disgust Higher accuracy for OA than YA faces for sadness No age-of-face effect for anger No own-age effect No age-of-face effects on mimicry
Malatesta et al., 1987a	14 OA	Photos of posed expressions	Happiness, anger, sadness, fear, neutrality	30 YA	Multiple choice emotion identification Personality test results of posers	Emotions rated in the neutral face were congruent with the posers' dominant trait emotions
Malatesta and Izard, 1984	10 YA, 10 MA, 10 OA, only females	Videos of women talking about biographic episodes (without sound)	Happiness, anger, sadness, fear, affection	30 adults (no specification of age)	MAX-Coding of facial expressions Multiple choice emotion identification	MAX-Coding: see Table 2 OA faces received less anger, but more sadness attributions than YA faces
Malatesta et al., 1987b	10 YA, 10 MA, 10 OA, only females	Videos of women talking about biographic episodes (without sound)	Sadness, fear, anger	10 YA, 10 MA, 10 OA, only females	Multiple choice emotion identification Rating of intensity of dominant emotion	No significant age-of-face effect on decoding accuracy Own-age bias in decoding accuracy Trend for more frequent attribution of sadness to OA than YA faces
Matheson, 1997	10 YA 10 OA, chronic pain patients	Videos of patients undergoing motion tests	Posed, masked and true pain, neutrality	39 YA, 24 OA	Rating of intensity of experienced pain	More pain attribution to OA, for all experimental conditions
Murphy et al., 2010	13 YA, 11 OA, only females	Videos of women talking about an imagined pleasant scenario (posed smile), pleasant biographical experience/response to winning a prize (spontaneous smile)	Posed smile, spontaneous smile	23 YA, 26 OA	Multiple choice rating of posed vs. spontaneous smile	Higher accuracy for YA than OA faces No own-age effect
Richter et al., 2011	4 YA, 4 OA, only females	Videos of women talking about biographic episodes (played with vs. without sound)	Happiness, anger, sadness	48 YA, 35 OA, only females	Continuous emotion rating, correlation of observers' and actors' ratings of felt emotions	Higher accuracy for YA than OA faces, no interaction with emotion Younger observers showed an own-age advantage when videos were played with, but not without sound
Riediger et al., 2011	58 YA, 56 MA, 57 OA	Still posed expressions [FACES developed by Ebner et al., 2010]	Happiness, anger, sadness, fear, disgust, neutrality	52 YA, 51 MA, 51 OA	Continuous emotion rating	OA faces were rated as more intense on inaccurate emotions, but as less intense on accurate emotions Own-age effect for happiness and anger Attribution of more inaccurate neutrality, anger, and sadness to OA than YA faces
Riediger et al., under review, Study 2	16 YA and 16 OA per smile type	Videos of adults watching amusing film clips (spontaneous smile), or posing smiles	Posed smile, spontaneous smile	48 YA, 49 OA	Multiple choice rating of posed vs. spontaneous positive-affect smile	More frequent attribution of positive-affect smile to OA than YA faces, effect was more pronounced for YA than OA observers Own-age advantage in decoding posed smiles for both YA and OA, and in decoding spontaneous smiles for OA No main effect of facial age on decoding accuracy

*YA, younger adults; MA, middle-aged adults; OA, older adults*;

1 *As Ebner et al. (2013) report identical emotion decoding data to Ebner et al. (2012), the former study is not mentioned in the table*.

### Expressivity

One possible explanation for reduced decoding accuracy for older faces may be that there actually is a difference in the way older and younger adults express emotions in their faces. Supporting this assumption, older adults performed worse than younger adults when following muscle-by-muscle instructions for constructing facial prototypes of emotional expressions (Levenson et al., 1991). Thus, due to age-related changes in flexibility and controllability of muscle tissue, the intentional display of facial emotions may become less successful with age and displays of unintended blended emotions may become more likely (Ebner et al., 2011c). In line with this assumption, observers more accurately judged whether videotaped speakers were telling the truth or lying when the speakers were older than when they were younger (Ruffman et al., 2012). Borod et al. (2004) further argued that an age-related decline in the frontal lobe may change emotional facial expressions, as frontal structures are especially important for the production of facial expressions and are highly vulnerable to aging.

Notably, these explanations may only account for age effects in posed expressions. For spontaneous expressions, results rather point to the assumption that younger and older adults do not differ in expressivity. In several studies, younger and older adults were filmed while reliving an emotional event or watching emotional film clips. Afterwards, their facial reactions were analyzed with objective coding systems such as FACS (Ekman et al., 1978), or MAX (Izard, 1979). An overview of these studies is given in Table 2. Although an early study suggested that older adults display more masked, that is, dissimulated, mixed and fragmented facial expressions than younger adults (Malatesta and Izard, 1984), later studies did not confirm these age differences in expressivity (Levenson et al., 1991; Tsai et al., 2000; Kunz et al., 2008), or even found higher expressivity for older faces (Malatesta-Magai et al., 1992). Thus, intentional displays of emotions may become less successful with age, but spontaneous emotional facial reactions seem to remain equally expressive throughout the life span. Besides, lower decoding accuracy for older faces cannot be fully explained by age differences in expressivity, because this effect has also been found when artificially created face stimuli controlled for expressivity were used (Hess et al., 2012).

**Table 2:** **Summary of research on age differences in facial expressivity**.

**Authors**	**Age-of-face**	**Emotion induction**	**Target emotions**	**Coding system**	**Other dependent variables**	**Main results**
Kunz et al., 2008	46 YA, 61 OA	Pressure stimulation, electrical stimulation	Pain	FACS (Ekman et al., 1978)	Self-reported pain	No age differences in facial expressions or self-reported pain
Levenson et al., 1991	20 OA, 62 YA	Muscle-by-muscle instruction for posing expressions Reliving biographical episodes	Happiness, anger, sadness, fear, disgust, surprise	FACS (Ekman et al., 1978)	Self-reported experienced emotions ANS activity	OA performed worse than YA when posing facial expressions and experienced the emotions to a lower degree than YA Spontaneous expressions and experiences of target emotions were comparable between YA and OA
Magai et al., 2006	32 YA, 32 MA, 32 OA	Reliving biographical episodes	Anger, sadness	MAX (Izard, 1979)	Self-reported experienced emotions	YA showed more shame, contempt and joy than OA OA showed more knitted brows than YA OA experienced more interest than YA and MA, no age differences for the remaining emotions Greater heterogeneity in experienced emotions in OA than YA, but this was due to age differences in chosen topics
Malatesta and Izard, 1984	10 YA, 10 MA, 10 OA, only females	Reliving biographical episodes	Happiness, anger, sadness, fear, affection	MAX (Izard, 1979)	Self-reported experienced emotions	OA showed more masked, mixed and fragmented partial expressions than YA OA showed more anger and contempt, less sadness than YA No age differences in experienced emotions
Malatesta-Magai et al., 1992	80 YA, 80 OA	Reliving biographical episodes	Anger, sadness, fear, interest, affection	MAX (Izard, 1979)	Self-reported experienced emotions	OA showed more anger, sadness, fear and interest than YA OA experienced more interest, no age differences for the remaining emotions
Tsai et al., 2000	48 YA, 48 OA	Watching emotional film clips	Sadness, amusement	Coding system by Gross and Levenson (1993)	Self-reported experienced emotions Cardiovascular response	No age difference in facial expressions No age difference in experienced emotions Smaller cardiovascular reactions in OA than YA

*YA, younger adults; MA, middle-aged adults; OA, older adults*.

Nevertheless, analyses of spontaneous facial expressions suggest that there may be age-related “dialects,” that is, slight differences in the way older and younger adults express certain emotions. For example, older adults expressed sadness mainly through a lowered head, whereas younger adults also showed lowered brows (Malatesta and Izard, 1984). While reliving anger and sadness eliciting episodes, younger adults showed longer durations of shame, contempt and joy expressions, which may be interpreted as a cynical, self-conscious, perhaps mocking facial presentation that is common in younger adults (Magai et al., 2006). Older adults, on the other hand, showed more knitted brows, possibly indexing a generalized distress configuration in a regulated form, serving to indicate that negative emotion is present, but protecting social partners from emotional contagion (Magai et al., 2006). Notably, these age differences were not related to a corresponding age difference in experienced emotions (Malatesta and Izard, 1984; Magai et al., 2006). However, it is unclear whether these differences are actually due to the participants' age, or whether these are cohort-specific differences. Thus, long-term studies examining several cohorts in different ages would be necessary to follow up this question.

### Age-related changes in the face

Decoding accuracy for older faces may also be reduced due to age-related changes in the face such as wrinkles and folds (see Albert et al., 2007; Porcheron et al., 2013; for overviews of age-related changes in the face). The wrinkles and folds in the older face may resemble emotional facial expressions and lead to the impression of a permanent affective state (Hess et al., 2008). These background affects may make older adults' facial expressions more ambiguous and reduce the signal clarity (Ebner and Johnson, 2009; Hess et al., 2012). Thus, when emotional expressions were rated on multiple intensity scales for target as well as non-target emotions instead of forced-choice scales, raters attributed less of the target emotions, but more non-target emotions to older faces (Riediger et al., 2011; Hess et al., 2012). Further supporting this account, no age-of-target effects emerged for decoding emotional prosody (Dupuis and Pichora-Fuller, 2011), suggesting that lowered decoding accuracy for older targets may be specific to faces.

These age-related changes in the face may also systematically bias emotional attributions. Hess et al. (2008) suggested that facial expressions and morphological features can have similar effects on emotional attributions (“functional equivalence hypothesis”). Thus, age-related changes in the face may both reduce the signal clarity and bias emotional attributions. Physiognomic features that are frequently found in older faces, such as for example down-turned corners of the mouth may be misinterpreted as emotional expressions. Supporting this assumption, older faces received more sadness attributions than younger faces (Malatesta and Izard, 1984).

However, so far it is unclear whether these effects are due to general aging effects *per se* (e.g., loss of muscle tone) or due to trace emotions (Malatesta et al., 1987a). Interestingly, emotions participants attributed to older senders' neutral expressions were congruent with senders' dominant trait emotions (Malatesta et al., 1987a). Thus, frequently experienced emotions may leave a trace on the face (“habitual emotional expressions”), so that in older age, the neutral expression resembles these emotions. However, to our knowledge, this result has not yet been replicated. Clearly, more research on the relationship between emotionality and age-related changes in the face is needed. Here, long-term studies may constitute a valuable extension of previous research.

### Emotion schemas

As an alternative explanation for reduced decoding accuracy for older faces, Ebner et al. (2011a) suggested that facial expression prototypes are more likely young faces. The authors argue that emotion schemas may be developed in childhood from the young faces of parents and TV and movie depictions of facial expressions, where older individuals are underrepresented (Signorielli, 2004). Thus, emotion schemas may be better calibrated to decode emotions in younger than older faces. A strong effect of the frequency of contact with faces of specific age groups has been confirmed for face identity recognition (Harrison and Hole, 2009). Furthermore, studies investigating the ability to discriminate among individual faces suggest that early in childhood, perceptual processes become tuned to adult faces as the faces children have been most frequently exposed to since birth (see Macchi Cassia, 2011, for a review). Thus, 3-year old children who had frequent contact with elderly people showed no processing advantage for younger over older adult faces, whereas non-experienced children did (Proietti et al., 2013). However, these processes may still be modulated by experience with faces of different age groups during adulthood (Macchi Cassia, 2011). As not only younger, but also older adults substantially differ in the amount of contact with older people (Wiese et al., 2012), emotion schemas for older faces may still vary in older observers.

Pertaining to decoding accuracy, this explanation still needs empirical investigation. Future studies could examine a sample with frequent contact with older adults during childhood, for example children that grew up in multi-generational homes. If this sample showed less difference in decoding accuracy between younger and older faces than a control group, this would support the hypothesis of less elaborated emotion schemas as an underlying mechanism. Furthermore, the influence of experience with older faces during early and late adulthood may be investigated by examining individuals of varying age and with varying amount of contact with older adults.

### Attitudes toward older adults

An alternative explanation may be that younger adults are preferred over older adults (Ebner and Johnson, 2009). Although there are both positive and negative elements in age stereotypes (e.g., Hummert et al., 2004; Kornadt and Rothermund, 2011), both younger and older adults showed more positive implicit attitudes (Ebner et al., 2011b) and explicit evaluations (Ebner, 2008) of younger than older faces. In addition, young adults implicitly associated themselves more closely with the concept of being young than old (Wiese et al., 2013b).

Furthermore, as individuals resort on stereotype knowledge about social groups when decoding ambiguous facial expressions of strangers (Hess and Kirouac, 2000), stereotypes may also, just like age-related changes in the face, bias the attribution of emotions. For example, if individuals hold the stereotype of older persons being less satisfied, they may be more prone to attribute sadness and less prone to attribute happiness to an older compared to a younger face. Higher decoding accuracy for emotions corresponding to stereotypes and lower decoding accuracy for emotions contradicting stereotypes may result. This may not that much apply to the posed expressions typically used in emotion decoding studies, which are rather unambiguous, but more to spontaneous expressions that we encounter in everyday life, which can be mixtures of several emotions, or be masked behind socially more desirable emotions. In line with this assumption, for spontaneous expressions, Hühnel et al. (2014) did not replicate the pattern of generally lower decoding accuracy for older faces. Instead, happiness and disgust were more accurately decoded in younger faces, whereas sadness was more accurately decoded in older faces. Also, the previously mentioned result that older faces received more sadness attributions (Malatesta and Izard, 1984) may not only be due to age-related changes in the face, but also to age-related stereotypes. In this vein, observers attributed more pain (Matheson, 1997), but less anger (Malatesta and Izard, 1984) to older faces. In addition, individuals displaying a happy facial expression were perceived as younger than individuals displaying a fearful, angry, disgusted or sad expression (Voelkle et al., 2012). In the same vein, Bzdok et al. (2012) found a negative association between the perceived age and happiness of faces. Although this pattern of results is somewhat mixed, it seems that youth is more likely associated with happiness, whereas older age is more likely associated with sadness.

However, aging stereotypes in emotional domains were not found in explicit measures, possibly because they are socially undesirable. When participants were directly asked to describe “typical” younger and older individuals, relatively neutral stereotypes in social and emotional domains were found (Boduroglu et al., 2006). Also, not all studies using spontaneous expressions found emotion-specific effects of the faces' age on decoding accuracy (Malatesta et al., 1987b; Richter et al., 2011). Furthermore, contradicting the assumed association between youth and happiness, Riediger et al. (under review) found a more frequent attribution of positive emotions to smiles shown by older compared to younger individuals. Clearly, more research is needed, using more subtle or implicit measures for age-related stereotypes, such as IAT (implicit association test), and relating them to attributed emotions.

### Visual scan patterns

There is some evidence that visual scan patterns may differ, depending on the age of the face that is being observed. Specifically, both younger and older observers looked longer at the eye region of older than younger neutral faces, and longer at the mouth region of younger than older neutral faces (Firestone et al., 2007). Considering the above mentioned higher importance of the eye region for expressions of anger, fear, and sadness, and the mouth region for sadness and disgust, one could expect higher decoding accuracy for younger than older faces for disgust and happiness, but not for anger, fear and sadness. However, among the studies examining age-of-face effects on decoding accuracy, only one was in line with this pattern (Hühnel et al., 2014). The majority of previous research found lower decoding accuracy for older faces, independent of the type of expression. Furthermore, other studies found that visual scan patterns were independent of the faces' age (He et al., 2011) or depended on the type of expression (Ebner et al., 2011c). Thus, Ebner et al. (2011c) only confirmed the result of longer looking at the eye region of older than younger faces for expressions of anger. For disgust, the opposite pattern with longer looking at the lower half of older than younger faces was found. There were no age differences for happy, fearful, sad or neutral faces. Thus, the result of different visual scan patterns for younger than older faces may not be generalizable across all facial expressions. In addition, whereas young observers' expression identification of young faces was better the longer they looked at the upper half of faces, older observers' expression identification of young faces was better the longer they looked at the lower half of faces (Ebner et al., 2011c). Thus, the assumption of one visual scan pattern leading to higher accuracy for both younger and older observers and younger and older faces might not always be appropriate.

Considering these mixed results, more research on this topic, relating visual scan patterns for faces with varying age and facial expressions to decoding accuracy is needed to decide whether visual scan patterns may account for age-of-face effects on decoding accuracy. So far, evidence rather contradicts the assumption of visual scan patterns as an underlying mechanism.

### Neural processing

To our knowledge, previous EEG studies only examined the neural processing of neutral, but not emotional younger and older faces (e.g., Wiese et al., 2008, 2012; Ebner et al., 2011b; Wolff et al., 2012). These studies revealed that the age of the face influenced both early and late ERP components (Ebner et al., 2011b), suggesting that age already influences early processing stages. For older faces, enlarged amplitudes of the N170, a negative deflection over occipito-temporal sites, have been found (Wiese et al., 2008, 2012), suggesting that structural encoding may be more difficult for older faces. Further, enlarged Late Positive Potentials (LPP, a positive deflection over parietal sites) for older faces suggest more controlled processing of older than younger faces (Ebner et al., 2011b). This latter assumption is further supported by recent fMRI results of greater dmPFC activation for older than younger emotional faces (Ebner et al., 2012). However, so far only very little research on neural processing of younger and older emotional faces has been conducted, allowing no definite conclusion on neural processing as an underlying mechanism. Thus, further research examining the relation of neural processing of emotional younger and older faces to decoding accuracy is needed.

## Own-age advantage

Apart from the above mentioned main effects of the ages of the observer and the face, age congruence between the observer and the face might influence decoding accuracy as well. As emotions are less accurately decoded in out-group than in-group faces (Thibault et al., 2006) and age is an important social category, one could expect an own-age advantage in face processing.

In line with this assumption, participants tended to look longer at own-age faces and longer looking at own-age faces predicted better own-age expression identification (Ebner et al., 2011c); they were more distracted by own-age faces (Ebner and Johnson, 2010) and fMRI-Studies report different activities for own-age than other-age faces (Wright et al., 2008; Ebner et al., 2011a, 2013), possibly indexing a preference for and more interest in own-age faces. Some EEG-studies report partly comparable own-age and own-race effects on ERPs for neutral faces (Wiese et al., 2008; Ebner et al., 2011b; but see Wiese, 2012; Wiese et al., 2013a, for partly different ERP correlates). Furthermore, several studies found that participants remembered own-age faces better than other-age faces (see Rhodes and Anastasi, 2012, for a meta-analysis). There are two main explanations for this latter finding. Firstly, social cognitive theories suggest that faces of out-group members are cognitively disregarded and more superficially processed than faces of in-group members (Sporer, 2001). Secondly, more experience or contact with members of the own age group may lead to higher perceptual expertise with own-age faces (Rhodes and Anastasi, 2012) and to higher familiarity with the expressive style of the own age group (Malatesta et al., 1987b). So far, evidence is more in line with the latter explanation, as the amount of contact appears to be related to face identity recognition (Harrison and Hole, 2009; Wiese et al., 2012, 2013b; Wolff et al., 2012) and facial expression decoding accuracy (Ebner and Johnson, 2009) of other-age faces. Hugenberg and colleagues (Hugenberg et al., 2010, 2013) suggested an integration of both theories in the Categorization- Individuation Model, which may also be useful to explain the own-age advantage. According to this model, own-group biases may be due to the combined influence of social categorization, the motivation to individuate and perceptual experience (Hugenberg et al., 2010). An overview of possible mechanisms underlying own-age effects on decoding accuracy is given in Figure 1 (central part).

It is likely to assume that these in-group effects in face processing also influence facial expression decoding. Usually, facial expressions of in-group members are more accurately decoded than expressions of out-group members, even if group membership is manipulated (Thibault et al., 2006; Young and Hugenberg, 2010). In addition, automatic affective responses to other persons' emotional expressions are congruent for ingroup members, but incongruent for outgroup members (Weisbuch and Ambady, 2008). In an early study, Malatesta et al. (1987b) confirmed an own-age advantage in facial expression decoding accuracy. In addition, Riediger et al. (under review) reported an own-age effect on the ability to differentiate between spontaneous and posed smiles. Surprisingly though, the majority of the extant research found no own-age advantage (Borod et al., 2004; Ebner and Johnson, 2009; Murphy et al., 2010; Ebner et al., 2011c, 2012, 2013; Hühnel et al., 2014) or an own-age advantage that was confined to specific emotions (Riediger et al., 2011). Thus, age congruence between the observer and sender of facial expressions seems to play a minor role for facial expression decoding, and the features that are important for identity recognition of faces may not be identical to those that are important for decoding facial expressions (Ebner and Johnson, 2009).

## Conclusions and future directions

To sum up, the age of the face seems to play an important role for the interpretation of facial expressions. Posed expressions are less accurately decoded in older compared with younger faces. However, for spontaneous expressions, results are rather mixed. As a possible explanation, older adults are less expressive when posing emotional expressions, but equally expressive when spontaneously showing emotions. Yet at the same time, age stereotypes and age-related changes in physiognomic features of the face may bias the attribution of certain emotions. Contrariwise, age congruence between observer and sender of facial expressions may only play a minor role for expression decoding.

Concerning the underlying mechanisms, more research is needed to decide which of the suggested mechanisms are likely to underlie age-of-face effects on decoding accuracy. Age differences in expressivity are unlikely to be the sole underlying mechanism, as age-of-face effects have also been found when expressivity was controlled for (Hess et al., 2012). Further, previous research rather argues against visual scan patterns as an underlying mechanism. For the remaining mechanisms, i.e., age-related changes in the face, emotion schemas, attitudes toward older adults, and neural processing, more research is needed to judge the applicability of these accounts. It is unlikely to assume only one single mechanism driving age-of-face effects. Rather, multiple mechanisms seem to affect decoding accuracy.

One of the aims of the present review was to outline promising areas of future research. Although several mechanisms have been proposed to underlie age-of-face effects on decoding accuracy, only very little research directly tested the influence of these mechanisms. Thus, in our view, the most important objective for future research in this area will be to directly examine the influence of each of these variables on decoding accuracy for younger and older faces. An overview of some interesting questions for future research is given in Box 1. For example, further EEG and fMRI studies would be suited to relate neural processing of younger and older emotional faces to decoding accuracy. In addition, long-term studies on age-related changes in the face and their relation to frequently experienced emotions, and on changes in emotion schemas, which may be related to contact frequency with different age groups, may shed further light on gradual evolvement of these mechanisms. Further research is also needed on the relationship between age-related stereotypes and decoding biases. Furthermore, so far the question whether age-of-target effects are specific for facial expressions, or whether they also emerge in other emotion channels, is not yet fully resolved, as there is only one previous study analyzing age-of-target effects on decoding emotional prosody (Dupuis and Pichora-Fuller, 2011). Finally, future studies may examine whether the lower decoding accuracy for older faces affects the quality of interpersonal interactions and relationships. Thus, we think that exploring age-of-face effects on facial expression decoding and the underlying mechanisms is a promising and interesting area for future research.

Box 1 **Questions for future research**

Are age-related dialects for facial expressions due to aging effects *per se* (such as changes in flexibility and controllability of muscle tissue), or due to cohort-specific differences (such as differences in display rules)?

Does the frequency of contact to older adults during childhood and adulthood modulate age-of-face effects on decoding accuracy?

Are age-related changes in facial physiognomic features that resemble certain emotions due to aging effects *per se* (such as loss of muscle tone) or due to frequently experienced emotions, leaving a trace on the face?

Are age-related response biases in emotion decoding tasks related to implicit and explicit stereotypes of aging? Is the lower decoding accuracy for older faces related to more negative attitudes toward older than younger adults?

Do visual scan patterns differ for younger and older emotional faces? If yes, might this effect explain age-of-face effects on decoding accuracy?

Are age-of-face effects on expression decoding related to differences in neural processing of younger and older emotional faces?

What is the time course of neural processing of age and emotional expression of a face?

Does the age of the target affect emotion decoding in other emotion channels than facial expressions, such as emotional prosody or body language?

Does the lower decoding accuracy for older faces affect the quality of interpersonal interactions and relationships for older adults?

### Conflict of interest statement

The authors declare that the research was conducted in the absence of any commercial or financial relationships that could be construed as a potential conflict of interest.
